# Estimating Sepsis Incidence Using Administrative Data and Clinical Medical Record Review

**DOI:** 10.1001/jamanetworkopen.2023.31168

**Published:** 2023-08-29

**Authors:** Lisa Mellhammar, Erik Wollter, Jacob Dahlberg, Benjamin Donovan, Carl-Johan Olséen, Per Ola Wiking, Norman Rose, Daniel Schwarzkopf, Marcus Friedrich, Carolin Fleischmann-Struzek, Konrad Reinhart, Adam Linder

**Affiliations:** 1 Department of Clinical Sciences, Division of Infection Medicine, Lund University, Lund, Sweden; 2 Institute of Infectious Diseases and Infection Control, Jena University Hospital, Jena, Germany; 3 Berlin Institute of Health, Campus Virchow-Klinikum, Berlin, Germany; 4 Stiftung Charité, Berlin, Germany; 5 Department of Anesthesiology and Operative Intensive Care Medicine (CCM, CVK), Charité Universitätsmedizin Berlin, Corporate Member of Freie Universität Berlin, Humboldt-Universität zu Berlin, Berlin, Germany

## Abstract

**Importance:**

Despite the large health burden, reliable data on sepsis epidemiology are lacking; studies using *International Statistical Classification of Diseases and Related Health Problems (ICD)*–coded hospital discharge diagnosis for sepsis identification suffer from limited sensitivity. Also, *ICD* data do not allow investigation of underlying pathogens and antimicrobial resistance.

**Objectives:**

To generate reliable epidemiological estimates by linking data from a population-based database to a reference standard of clinical medical record review.

**Design, Setting, and Participants:**

This was a retrospective, observational cohort study using a population-based administrative database including all acute care hospitals of the Scania region in Sweden in 2019 and 2020 to identify hospital-treated sepsis cases by *ICD* codes. From this database, clinical medical records were also selected for review within 6 strata defined by *ICD* discharge diagnosis (both with and without sepsis diagnosis). Data were analyzed from April to October 2022.

**Main outcomes and measures:**

Hospital and population incidences of sepsis, case fatality, antimicrobial resistance, and temporal dynamics due to COVID-19 were assessed, as well as validity of *ICD-10* case identification methods compared with the reference standard of clinical medical record review.

**Results:**

Out of 295 531 hospitalizations in 2019 in the Scania region of Sweden, 997 patient medical records were reviewed, among which 457 had sepsis according to clinical criteria. Of the patients with clinical sepsis, 232 (51%) were female, and 357 (78%) had at least 1 comorbidity. The median (IQR) age of the cohort was 76 (67-85) years. The incidence of sepsis in hospitalized patients according to the Third International Consensus Definitions for Sepsis and Septic Shock (Sepsis-3) criteria in 2019 was 4.1% (95% CI, 3.6-4.5) by medical record review. This corresponds to an annual incidence rate of 747 (95% CI, 663-832) patients with sepsis per 100 000 population. No significant increase in sepsis during the COVID-19 pandemic nor a decrease in sepsis incidence when excluding COVID-19 sepsis was observed. Few sepsis cases caused by pathogens with antimicrobial resistance were found. The validity of *ICD-10*–based case identification in administrative data was low.

**Conclusions and Relevance:**

In this cohort study of sepsis epidemiology, sepsis was a considerable burden to public health in Sweden. Supplying administrative data with information from clinical medical records can help to generate reliable data on sepsis epidemiology.

## Key Points

**Question:**

What is the population-based sepsis incidence estimated from administrative data and clinical medical record review?

**Findings:**

This cohort study of 457 patients with sepsis generated reliable data on sepsis epidemiology, including pattern of antimicrobial resistance and temporal dynamics due to COVID-19, by linking administrative data from hospital information systems to a reference standard of clinical medical record review.

**Meaning:**

These findings suggest sepsis was a considerable burden to public health and that this study design may be used in different settings and thereby may generate high-quality and comparable data that could identify areas for improvement in sepsis care.

## Teaser

This cohort study estimates the population-based sepsis incidence in Sweden using administrative data and clinical medical record review.

## Introduction

Despite the large health burden of sepsis, there is a substantial lack of reliable, population-level data on its epidemiology.^1,2^ On the one hand, this may be because data are often generated by prospective, multicenter studies for which a population denominator is difficult to derive. On the other hand, studies^3^ covering larger populations, such as those of 1 region or country, often use administrative data by identifying patients by *International Statistical Classification of Diseases and Related Health Problems, Ninth Revision (ICD-9)* and *Tenth Revision (ICD-10) *codes with poor and variable precision to identify clinical sepsis cases. This imprecision is mainly caused by a lack of sensitivity in case identification, which leads to a considerable underestimation of sepsis cases in administrative data (*ICD*-coded hospital discharge diagnoses).^1,4,5,6,7,8^ In addition, administrative data do not allow investigation of underlying pathogens and patterns of antimicrobial resistance, which limits our understanding of temporal dynamics and causative pathogens in sepsis. This particularly emerged during the COVID-19 pandemic, as a considerable proportion of patients with COVID-19 experienced sepsis but may not have been coded specifically with sepsis.^9,10^ Therefore, the World Health Organization called for improved data on sepsis epidemiology and emphasized clinical medical record review as the reference standard.^11^ However, medical record review is often resource-intensive to conduct, as the hospital incidence of sepsis is comparably low (around 6%) and it may require large amounts of medical records to be reviewed to generate reliable data on the burden of sepsis or specific sampling strategies.^5^

The primary aim of this study is to generate reliable population-level data on the burden of sepsis by supplying a population-based database with information from clinical medical record review. Secondarily, we aimed to analyze temporal trends of sepsis incidence during the COVID-19 pandemic and to assess outcomes of antimicrobial resistance on sepsis incidence.

## Methods

We conducted a retrospective, observational cohort study, linking a population-based database of all acute hospitalizations of the Scania region in Sweden with information from clinical medical records to investigate the epidemiology of sepsis. Ethical permission was granted by the Swedish Ethical Review Agency with a waiver of informed consent because the research involved minimal risk and only used data from patient records. The Strengthening the Reporting of Observational Studies in Epidemiology (STROBE) reporting guidelines were followed.^12^

### Setting, Data, and Procedure

We used the medical records database of the Scania region from 2019 and 2020. Among all patients hospitalized in 2019 and 2020 in the Scania region, a disproportional, stratified, random sampling was drawn for medical record review with the purpose to achieve a sufficient number of patients with sepsis. The sampling strata were defined according to *ICD* codes. *ICD* codes are manually applied by the discharging physician at the time of hospital discharge. Sepsis should be coded by R codes for sepsis (R651 or R572), although A codes and B codes for sepsis are sometimes used (eg, A021, A327, A403, or B377).^13,14^ These explicit sepsis codes may underestimate the incidence of sepsis, which is why another approach to identify sepsis is necessary, which is to combine codes for infection and organ dysfunction (implicit sepsis case identification). Patient medical records were randomly selected from 6 mutually exclusive categories by the following *ICD*-coded hospital discharge diagnoses in the administrative health database: patients with explicit sepsis (R codes), explicit sepsis (A codes), implicit sepsis, infection without organ dysfunction, and organ dysfunction without infection, as well as patients without infection, organ dysfunction, or sepsis. Extraction groups are depicted in the flowchart in the Figure.

**Figure.**
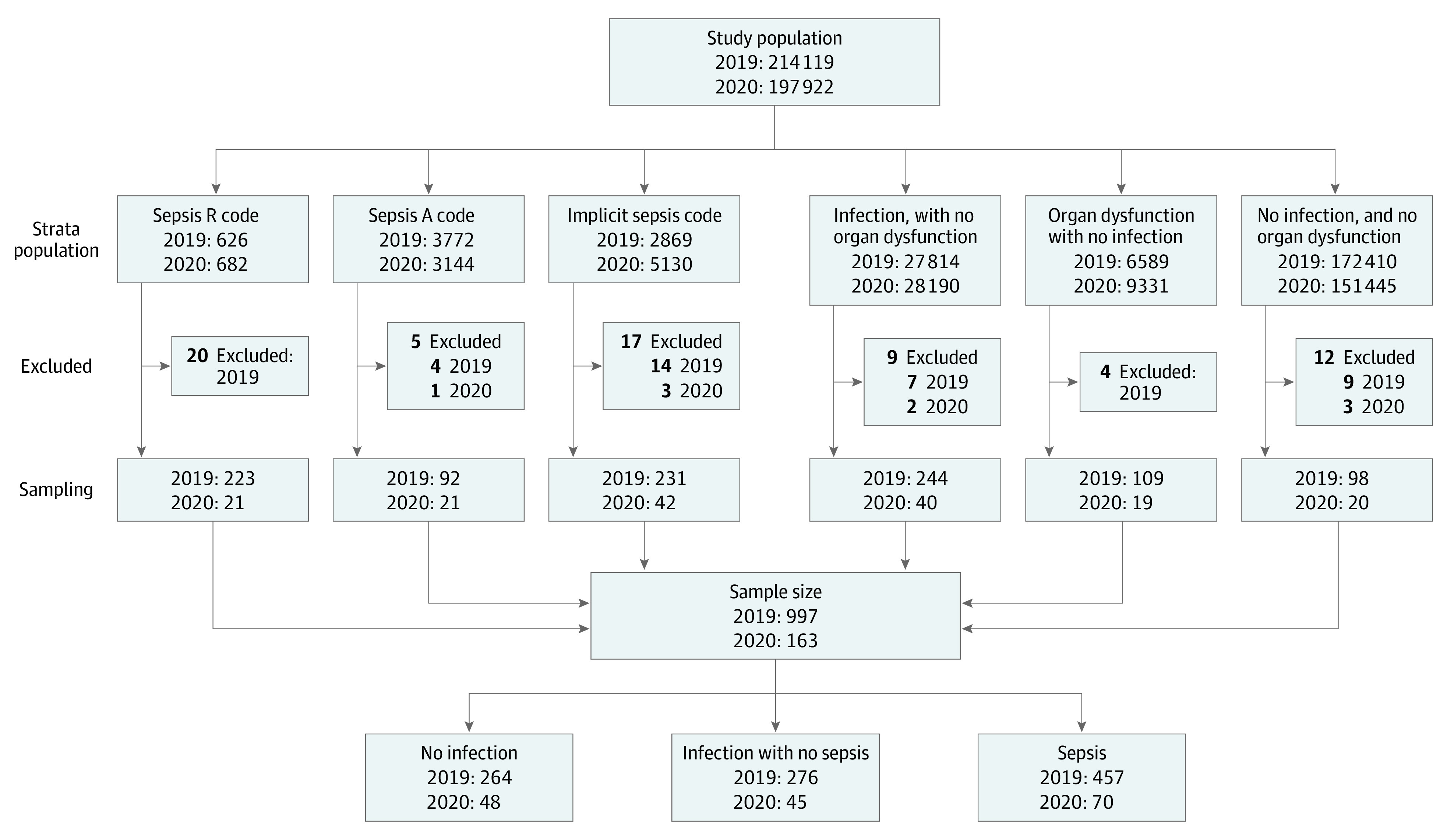
Schematic of Study Extraction and Design

We aimed for including 1000 patients in 2019 for medical record review, and extraction was disproportionate to capture more sepsis cases: 23% patients with R-coded sepsis, 23% patients coded with implicit sepsis, 23% patients with a code for infection but no code for organ dysfunction or sepsis, and 10% from each of the other extraction groups. Details on definitions for different *ICD* groups are included in the eAppendix in Supplement 1. Data were primarily extracted and analyzed for 2019, but measures were repeated for data from 2020 to investigate the sepsis incidence during the COVID-19 pandemic. For 2020, we aimed for including 160 patients for medical record review: 26% were patients coded with implicit sepsis, 26% were patients with a code for infection but no code for organ dysfunction or sepsis, and 13% were from each of the other extraction groups.

### Estimates According to Administrative Data in the Medical Record Database

We identified patients with sepsis by different *ICD*-*10* codes or combinations coded as primary or secondary discharge diagnosis in the medical record database: (1) R Codes, (2) A Codes, (3) explicit coding (A or R), and (4) implicit coding (infection and organ dysfunction). Patients identified with sepsis by *ICD* case identification strategies are denoted as the *ICD*-coded sepsis subgroup in the remainder of the article.

### Medical Record Review

Medical record reviews were primarily conducted by medical researchers (E.W., J.D., B.D., C.J.O., P.O.W.) who had received proper training to perform the task using a structured protocol. The collected data were also validated by an infectious diseases physician (L.M.), and if the classification differed, a second infectious diseases physician classified the patient (A.L.). Sepsis and septic shock were defined according to the Third International Consensus Definitions for Sepsis and Septic Shock (Sepsis-3) definitions.^14^ Sepsis-3 criteria were modified to be applicable outside intensive care units (ICUs) (eTable 1 in Supplement 1).^14^ Patients with sepsis identified by medical record review are denoted as the clinical sepsis subgroup in the remainder of the article.

Organ dysfunction was defined as an increase in total sequential organ failure assessment (SOFA) of 2 or more. A time window of 30 hours was used for SOFA calculation to maximize the chances of obtaining a complete SOFA, including vital parameters and morning blood samples. SOFA score was calculated repeatedly, and the time window with the highest change in SOFA was chosen. Partial pressure of oxygen in arterial blood (Pao_2_) was preferably used. When not available, the oxygen saturation by pulse oximetry (Spo_2_) was used for values less than 96% and Pao_2_ was calculated by the Severinghaus equation.^15,16^ The Glasgow Coma Scale (GCS) was preferably used to quantify the central nervous system SOFA if registered in medical records. If GCS was unavailable, reaction level scale, which has been validated to correlate with GCS, was used.^17,18^ We regarded missing values in the SOFA as within the range of values from adjacent days, since this is in accordance with clinical management and a common approach in retrospective sepsis studies. If values were still missing, it was regarded as normal. There is no reference standard for infection definition. We used the newly published Linder-Mellhammar Criteria of Infection (eTable 2 in Supplement 1).^19^

Bacterial isolates were identified at the clinical microbiology department (Laboratory Medicine Skåne, Lund, Sweden). Definitions of susceptibility were characterized according to European Committee on Antimicrobial Susceptibility Testing (EUCAST) guidelines.^20^ Infections were deemed to be caused by an identified bacteria if growth of a pathogen was demonstrated in blood or from the suspected site of infection. The following resistant pathogens were registered: methicillin-resistant *Staphylococcus aureus* (MRSA), penicillin-resistant pneumococci, β-lactam–resistant *Haemophilus influenzae,* enterobacterales and acinetobacter with resistance caused by extended spectrum β-lactamases (ESBL), metallo-β-lactamase–producing *Pseudomonas aeruginosa,* and enterococci with resistance to vancomycin. The following comorbidities were recorded according to Charlson Comorbidity Index: myocardial infarction, congestive heart failure, cerebrovascular accident, peripheral vascular disease, connective tissue disease, liver disease, peptic ulcer disease, hemiplegia, solid tumor, leukemia, lymphoma, AIDS, chronic obstructive pulmonary disease, dementia, diabetes, and chronic kidney disease.^21^

### Statistical Analysis

The data were analyzed in SPSS statistical software version 27 (IBM) with complex data analysis module. Data were analyzed between April and October 2022. To compensate for the disproportionate undersampling and oversampling, participants were assigned study weights depending on the population sizes of the extraction strata they belonged to. Study participants with duplicate entrants and participants not resident in the Scania region were excluded from the analysis. The weights were also adjusted according to excluded patients who did not reside in the region. The baseline characteristics of the sample population, the *ICD*-coded sepsis subgroup, and the clinical sepsis subgroup were calculated using the weighted frequencies with 95% CIs for categorical variables, while weighted medians and their IQRs were used for continuous variables. Weighted case fatality rates were calculated using complex frequency data analysis. The sensitivity, specificity, positive predictive value, and negative predictive value were calculated using complex sample-weighted cross-tabulation for sepsis population identified by *ICD* codes in administrative data, and clinical sepsis reference standard population. Cumulative incidence was calculated using complex sample weighted frequency. Cumulative incidence was converted into incidence rate and its 95% CI using the following equation: 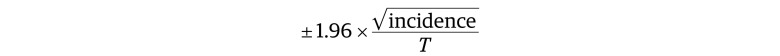
where *T* denotes person-years at risk, and the total study population and the mean Scania adult population of 1 076 035 in 2019 and 1 091 660 in 2020 extracted from official Region Scania population.^22^ Incidences adjusted for sampling weights were compared using χ^2^ test. Significance was set at *P*<.05 and all tests were 2-sided.

## Results

### Study Inclusion and Baseline Sample Characteristics

Among all acute hospitalizations in Scania (295 531 in 2019 and 217 246 in 2020) in 214 119 patients in 2019, and 197 922 patients in 2020, we identified 4398 patients with sepsis in 2019 and 3826 in 2020 by explicit *ICD* codes. From the database, 997 patients were selected for clinical medical record review in 2019, among whom 457 had sepsis according to clinical criteria. Of the patients with clinical sepsis, 232 (51%) were female, and 357 (78%) had at least 1 comorbidity. The median (IQR) age of the cohort was 76 (67-85) years. Flow of inclusion is depicted in the Figure. Baseline characteristics of the clinical medical record–reviewed sample population (total sample population, sepsis subpopulation identified by explicit *ICD* codes in the sample population, and clinical sepsis population) are presented in Table 1.

**Table 1.** Baseline Characteristics of the Study Population, Explicit Sepsis *ICD-10* Group, and Sepsis-3 Reference Standard Subgroup^a^

Factor	Patients, % (95% CI)		
	Sample population (N = 997)	Sepsis-*ICD-10* subgroup (n = 315)	Sepsis-3 Subgroup (n = 457)
Age, median (IQR), y	71 (60-82)	75 (64-86)	76 (67-85)
Sex			
Female	46.5 (38.1-55.0)	39.6 (31.2-48.7)	50.8 (43.3-58.3)
Male	53.5 (49.1-58.3)	60.3 (51.7-68.9)	49.2 (42.8-55.7)
Admitted from nursing home	6.8 (3.6-12.3)	9.5 (5.4-16.1)	13.6 (9.2-19.6)
Discharged to nursing home	7.8 (4.4-13.6)	6.9 (3.4-13.5)	14.0 (9.3-20.7)
Intermediate care	17.0 (11.6-24.3)	23.8 (16.9-32.3)	27.7 (21.4-34.9)
Intensive care	5.6 (2.8-10.8)	20.9 (15.1-28.2)	15.6 (11.5-20.8)
Length of stay, median (IQR), d	3 (2-6)	9 (4-15)	6 (2-11)
Comorbidities			
Myocardial infarction	10.2 (6.2-16.3)	11.6 (7.0-18.5)	19.2 (13.9-25.9)
Peripheral vascular disease	2.6 (1.1-6.6)	6.9 (3.5-13.0)	6.6 (3.8-11.3)
Congestive heart failure	4.7 (2.4-8.7)	13.8 (8.7-21.1)	17.1 (12.1-23.6)
Mild diabetes	14.9 (9.9-22.0)	23.5 (16.7-32.1)	25.1 (19.0-32.4)
Severe diabetes	0.6 (0.4-1.0)	5.4 (2.5-11.3)	4.7 (2.5-8.8)
Mild liver disease	1.1 (0.2-5.2)	2.2 (0.7-7.1)	2.4 (0.9-6.5)
Severe liver disease	0.2 (0.1-0.4)	0.2 (0.1-0.5)	0.6 (0.1-2.6)
Chronic obstructive pulmonary disease	6.3 (3.4-11.3)	11.0 (6.5-18.0)	15.2 (10.5-21.5)
Chronic kidney disease stage 5	1.5 (0.5-4.7)	6.6 (3.3-13.0)	5.8 (3.2-10.4)
Connective tissue disease	3.2 (1.3-7.9)	7.6 (3.9-14.0)	9.0 (5.4-14.7)
Peptic ulcer disease	1.3 (0.3-4.8)	5.5 (2.6-11.4)	2.4 (1.2-4.7)
Cerebrovascular accident	11.2 (6.9-17.5)	20.9 (14.3-29.4)	20.3 (15.0-26.9)
Hemiplegia	1.3 (0.4-4.8)	4.1 (1.7-9.9)	2.9 (1.4-6.1)
Dementia	6.1 (3.1-11.8)	4.5 (1.9-10.0)	9.7 (6.1-15.0)
Solid tumor	11.8 (7.3-18.4)	16.9 (11.0-24.9)	14.0 (9.5-20.2)
Metastasis	4.6 (1.8-11.3)	6.6 (3.1-13.4)	10.5 (6.0-17.9)
No comorbidities	51.8 (43.3-60.2)	25.3 (18.3-33.9)	21.8 (16.1-28.8)

Abbreviation: *ICD-10, International Statistical Classification of Diseases and Related Health Problems, Tenth Revision.*

^a^ The weighted baseline demographic characteristics of the study sample population, sepsis-3 categorized subgroup, and explicit sepsis *ICD-10*-subgroup (A- and R-sepsis).

The baseline characteristics of sepsis populations identified by *ICD* codes and by patient medical record review (clinical sepsis) were mostly comparable without significant differences of clinical relevance except for a smaller proportion of female patients in the sepsis *ICD*-coded population. Most common comorbidities among patients with sepsis of both groups were diabetes, cerebrovascular accident, and myocardial infarction.

### Incidence of Sepsis

In 2019, the incidence of sepsis among hospitalized patients was 4.1% (95% CI, 3.6%-4.5%) identified by patient medical record review. In administrative data, the incidence for explicit sepsis and implicit sepsis code abstraction strategies yielded lower estimates of 1.0% (95% CI, 1.0%-1.1%) and 1.4% (95% CI, 1.4%-1.5%), respectively (Table 2). Extrapolating the results of medical record review to the population level, we found an incidence rate of 747 patients with sepsis per 100 000 population (95% CI, 663-832). Septic shock defined in accordance with Sepsis-3 by medical record review had a hospital incidence of 0.2% (95% CI, 0.2%-0.2%), and an incidence rate of 39 patients with septic shock per 100 000 population (95% CI, 32-48).

**Table 2.** Incidence of Sepsis According to Different Sepsis Definitions^a^

Parameter	Patients, % (95% CI)			
	Sepsis-3	R-code sepsis	Explicit sepsis^b^	Implicit, including explicit sepsis
Cumulative incidence^c^	4.1 (3.6-4.5)	0.2 (0.2-0.2)	1.0 (1.0-1.1)	1.4 (1.4-1.5)
Annualized incidence-rate per 100 000 person-years	747 (663-832)	46 (32-58)	287 (241-335)	401 (362-440)

^a^ The calculated incidence among hospitalized patients for Sepsis-3 reference standard and different *International Statistical Classification of Diseases and Related Health Problems, Tenth Revision–*code abstraction strategy in 2019.

^b^ Explicit sepsis includes A- and R-sepsis.

^c^ Cumulative incidence was calculated through weighted frequency.

### Thirty-Day and 90-Day Case Fatality Rates of Sepsis

The 30-day and 90-day all-cause case fatality rates of different sepsis definitions are presented in Table 3. Patients identified with explicit sepsis codes in administrative data had higher case fatality rates compared with patients with sepsis identified in medical record review. The accuracy of different *ICD* code abstraction strategies against the reference standard of clinical medical record review is presented in Table 4.

**Table 3.** The 30-Day and 90-Day All-Cause Case Fatality Rates of Different Sepsis Definitions^a^

Parameter	Case fatality rate, % (95% CI)			
	Sepsis-3 (n = 457)	R-code sepsis (n = 223)	Explicit sepsis (n = 315)^b^	Implicit, including explicit sepsis (n = 546)
30-d	15.5 (11.4-20.7)	29.1 (23.5-35.5)	18.9 (12.9-26.7)	17.0 (12.2-21.8)
90-d	20.1 (15.2-26.0)	33.2 (27.3-39.7)	23.2 (16.5-31.4)	24.2 (16.7-31.6)

^a^ The calculated weighted 30-day and 90-day case fatality rates for Sepsis-3 reference standard and for each code abstraction method used.

^b^ Explicit sepsis includes A- and R-sepsis.

**Table 4.** Sensitivity, Specificity, Negative Predictive Value, and Positive Predictive Value of Different *International Statistical Classification of Diseases and Related Health Problems, Tenth Revision* Code Abstraction Strategies^a^

Parameter	Coding performance, % (95% CI)		
	R-code sepsis	Explicit sepsis^b^	Implicit, including explicit sepsis
Sensitivity	4.1 (3.6-4.7)	25.3 (21.8-29.5)	35.3 (31.3-39.4)
Specificity	96.1 (94.5-98.0)	90.9 (89.7-92.2)	68.9 (63.5-74.3)
Positive predictive value	89.7 (87.1-92.3)	84.4 (79.1-89.7)	69.2 (68.1-70.1)
Negative predictive value	95.5 (94.9-96.1)	96.5 (95.9-97.0)	82.4 (81.5-83.4)

^a^ The calculated sensitivity, specificity, PPV, and NPV depending upon the *International Statistical Classification of Diseases and Related Health Problems, Tenth Revision–*code abstraction strategy.

^b^ Explicit sepsis includes A- and R-sepsis.

### Incidence and Case Fatality of Sepsis During the Pandemic

Incidence of sepsis in hospitalized patients according to Sepsis-3 criteria including sepsis due to COVID-19 was 6.7% (95% CI, 5.0%-9.0%). Excluding COVID-19 cases, the hospital incidence of sepsis was 4.5% (95% CI, 3.1%-6.6%) corresponding to a population-level incidence of 815 per 100 000 person-years (95% CI, 581-1047). There was no significant difference in incidences between 2019 and 2020, neither regarding all sepsis cases (χ^2^_1_ = 1.69; *P* = .19), nor sepsis without COVID-19 (χ^2^_1_ = 0.19; *P* = .79). The mortalities were higher during the pandemic; the 30-day case fatality rate of sepsis was 30.0% (95% CI, 17.2%-42.8%; *P* = .01) and the 90-day case fatality rate of patients with sepsis was 34.3% (95% CI, 20.6%-48.0%; *P* = .01).

### Antimicrobial Resistance

In the 997 patients with clinical medical records reviewed for 2019, 25 patients had bacteria with antimicrobial resistance in microbiological samples, 14 of those had an infection, and 11 of those had sepsis caused by bacteria with antimicrobial resistance. When extrapolating the results of medical record review to a population level, 17 per 100 000 population (95% CI, 5-29) had sepsis with bacteria with antimicrobial resistance (2.3% of sepsis cases). Among the pathogens with antimicrobial resistance, ESBL-producing enterobacterales were the most common cause of sepsis (7 patients). The second most common antimicrobial-resistant bacteria was β-lactam–resistant *Haemophilus influenzae,* causing sepsis in 3 patients. MRSA only caused 1 sepsis case. The 30-day case fatality rate of sepsis caused by antimicrobial-resistant pathogens was 24.5% (95% CI, 17.4%-31.4%).

## Discussion

By linking data from medical record review with administrative data of a population-based database, we were able to both assess the hospital incidence of sepsis and to present adjusted estimates for the population-level incidence of sepsis. We found that the incidence of clinical sepsis was 4.1% among hospitalized patients, or 747 per 100 000 population in 2019 in the region of Scania in Sweden. We could see an increase of sepsis during the pandemic although not significant, and no decrease when excluding sepsis due to COVID-19. Antimicrobial-resistant bacteria were the cause of sepsis in 17 per 100 000 population, or 2.3% of sepsis cases, in this fairly low antimicrobial-resistant prevalence (5%-10%) setting.^23^

The incidences are in the same range as presented by Rhee et al^2^ who found sepsis present in 6% of all adults’ hospitalizations, corresponding to a sepsis incidence of 710 per 100 000 population when analyzing electronic health record data with clinical medical record review validation. A comparison of population-level estimates is complicated by the fact that only a few population-level prospective studies exist. In the Faroe Islands, all community-acquired severe patients with sepsis aged older than 15 years were registered, measuring 644 per 100 000 population.^24^ The first global report on epidemiology of sepsis estimated the incidence to 678 per 100 000 population but with large variations between countries.^25^ Differences between incidences in studies may also be caused by variation in data sources and designs or differences in sepsis definitions than actual variations. These factors can also explain differences in mortality. Contrary to other studies, we did not observe a significant increase in sepsis during the COVID-19 pandemic, nor a decrease in sepsis incidence when excluding COVID-19 sepsis.^9,26^

In line with previous literature, only a minority of patients with sepsis received an explicit *ICD* code for sepsis, resulting in a low sensitivity of the sepsis case identifications that are used in many epidemiological studies.^1,4,7,27^ This may be because sepsis may not be diagnosed, mentioned in discharge letters, or coded as hospital discharge diagnosis.^2^ In a review and meta-analysis of *ICD* codes for sepsis identification, Liu et al^7^ demonstrated such low sensitivity but also showed a large heterogeneity. Factors that have been shown to affect accuracy of *ICD* codes for sepsis include ICU admission, severity of disease, community-acquired or nosocomial sepsis, the site of infection, and temporary trends.^2,28,29,30,31^ This was also true in this study, with more patients in the ICU receiving an *ICD* code for sepsis than patients outside the ICU, and a higher mortality among patients with an *ICD* code for sepsis despite fewer comorbidities. The reasons for this could be intensive care physicians more commonly caring for patients with sepsis or that the more severely ill ICU patients are more often regarded as having sepsis.

We included clinical data on antimicrobial resistance. We found that there were few cases of sepsis caused by pathogens with antimicrobial resistance. Epidemiological data on antimicrobial resistance are often routinely generated from microbiological laboratory isolates.^23^ Even though microbiological laboratory data are important for surveillance of antimicrobial resistance, the effect of antimicrobial resistance on the burden of sepsis is not well understood. Antimicrobial resistance might be more or less common in microbiological isolates than in clinical sepsis, which is why clinical data are important.^32,33^ Decisions regarding antimicrobial therapy in patients and policies should take clinical data into account. With the increasing burden of antimicrobial resistance, the incidence and mortality of sepsis and severe infections are likely to increase over time. At the same time, sepsis is an indication for broad-spectrum antimicrobial use, which risks increasing antimicrobial resistance further. Valid data on the outcomes of antimicrobial resistance in sepsis is needed since inaccurate data can result in adequate but unnecessary broad-spectrum antimicrobial treatment.^34^ There is an ongoing debate on how to measure reliable estimates of burden of sepsis and infections with antimicrobial-resistant pathogens.^32^ Cassini et al^33^ constructed a data model considering large amounts of both microbiological and clinical data. The model simulated estimations of the burden of disease of antimicrobial resistance. Such models rely on assumptions and extrapolations that may be limited by input data and are necessary to validate. For Sweden, the study by Cassini et al^33^ estimated the mortality in infections by antimicrobial resistance to 1.9 per 100 000 population, which is in line with the results from this study.

### Strengths and Limitations

The major strength of this study is the clinical medical record review and sample selection by a population-level database. Another strength of our study is the use of a clinically widely applicable definition of infection.^19^ The sepsis definitions have stated criteria for organ dysfunction but not for infection, and with variable definitions of infection, patients will be classified differently.^14^ At present, many different definitions for the infection component in sepsis research are used, making comparisons between studies difficult.^5,35,36,37^ The Linder-Mellhammar Criteria of infection are a validated criteria of infection that are intended for sepsis research, in and outside of the ICU.^19^ Other strengths are the inclusion of the outcomes of antimicrobial resistance and validation of 2 consecutive years, of which 1 was during the pandemic.

There are also limitations to consider. The 2020 sample was drawn to generate additional estimates on the outcomes of the COVID-19 pandemic. As it was an add-on to the original 2019 study, resources were too limited to draw a sample of the same size as in 2019. Furthermore, we used databases of 1 selected region in Sweden, yet it is a region which is considered representative for the whole country. Third, the accuracy of coding may differ in subgroups affecting the adjusted incidences. Fourth, the approach we present requires the availability of a population-based database that is linkable to patient medical records to be replicated in other regions or countries; however, such a database may not exist in many countries.

## Conclusions

This study demonstrates that sepsis is a considerable burden to public health in Sweden. Supplying administrative data with information from clinical medical records can help to generate more reliable data on sepsis epidemiology. Knowledge on epidemiology can improve care and is of importance for effective use of health resources.

## Notes

Supplement 1. **eAppendix.** ICD Codes Defining Sepsis, Infection, and Organ Dysfunction **eTable 1. **SOFA Score Adapted for Use Outside of Intensive Care **eTable 2. **The Linder-Mellhammar Criteria of Infection

Supplement 2.
Data Sharing Statement
