# Supplementary material for: Estimating Sepsis Incidence Using Administrative Data and Clinical Medical Record Review
Source: JAMA Netw Open. 2023 Aug 29;6(8):e2331168. doi: 10.1001/jamanetworkopen.2023.31168 (PMC10466163; doi:10.1001/jamanetworkopen.2023.31168)
Supplement: Supplement 1. — eAppendix. ICD Codes Defining Sepsis, Infection, and Organ Dysfunction eTable 1. SOFA Score Adapted for Use Outside of Intensive Care eTable 2. The Linder-Mellhammar Criteria of Infection [file jamanetwopen-e2331168-s001.pdf]

Supplemental Online Content

Mellhammar L, Wollter E, Dahlberg J, et al. Estimating sepsis incidence using administrative data and clinical medical record review in Sweden, 2019 to 2020. *JAMA Netw Open*. 2023;6(8):e2331168. doi:10.1001/jamanetworkopen.2023.31168

eAppendix. ICD Codes Defining Sepsis, Infection, and Organ Dysfunction
eTable 1. SOFA Score Adapted for Use Outside of Intensive Care
eTable 2. The Linder-Mellhammar Criteria of Infection

This supplemental material has been provided by the authors to give readers additional information about their work.

© 2023 Melhammar L et al. *JAMA Network Open.*

eAppendix. ICD Codes Defining Sepsis, Infection, and Organ Dysfunction

		ICD-10
Sepsis	Explicit sepsis codes	A02.1
		A20.0
		A20.7
		A21.7
		A22.7
		A24.1
		A26.7
		A28.2
		A32.7
		A39.1
		A39.2
		A39.3
		A39.4
		A40.
		A41.
		A42.7
		A48.3
		A49.9
		A54.8
		B00.7
		B37.6
		B37.7
		B49
		O75.3
		O85
		R65.0
		R65.1
		R57.2
	Severe sepsis	R65.1
	Septic shock	R57.2
Infections		J01
		J02
		J03
		J04
		J06
		J05
		J09
		J10
		J11
		J12
		J13
		J14

© 2023 Melhammar L et al. *JAMA Network Open.*

J15
J16
J17
J18
J20
J21
J22
J44.0
J44.1
J86
J85
A15
A16
U69.00
A36
A37
B38
B39
P23
P24.0
P24.8
P24.9
A00
A01
A02
A03
A04
A05
A06
A07
A08
A09
K35
K37
K36
K5702
K5703
K5712
K57.13
K57.22
K57.23
K57.32
K5733
K5742
K5743
K5752
K5753
K5782
K5783

© 2023 Melhammar L et al. *JAMA Network Open.*

K5792
K5793
K61
K65
K67
K63.0
K63.1
K75.0
K75.1
K81.0
K77.0
U69.40!
P38
P77
P78.1
A46
B47
L03
L04
L08
L05
B00
B07
B08
B09
H05.0
H60.2
H70.0
J36
J39.0
J39.1
L02
N10
N15.1
N15.9
N34
N30
N39.0
N41
N45
N48.2
N49
N70
N71
N72
N73
N74*
N75
N76

© 2023 Melhammar L et al. *JAMA Network Open.*

N77
N61
N98.0
A59
A55
A56
A39
G00
G01
G02
G03
G04
G05
G06
G07
G08
A17
A81
A83
A84
A85
A86
A87
A88
A89
I32
I33
I39
I40
I41
I80
I38
I98.1
T82.6
T82.7
T83.5
T83.6
T84.5
T84.6
T84.7
T85.7
A18
A19
A20
A21
A22
A23
A24
A25

© 2023 Melhammar L et al. *JAMA Network Open.*

A26
A27
A28
A32
A38
A42
A43
A44
A48
A49
A54
A69.0
A69.1
A69.2
A69.8
A69.9
A90
A91
A97
B37
B40
B41
B42
B43
B44
B45
B46
B48
B49
B50
B51
B52
B53
B54
M00
M01
M86
T80.2
T81.4
T88.0
A50
A65
A74
A75
A77
A78
A79
A80
A92

© 2023 Melhammar L et al. *JAMA Network Open.*

A93
A94
A95
A96
A98
A99
B01
B02
B03
B04
B05
B06
B25
B26
B27
B33
B34
B55
B58
B60
B64
B67
B95
B96
B97
B98
B99
P35
P37
P39
O75.3
O85
O030
O035
O040
O045
O050
O055
O060
O065
O070
O075
O08.0
O86
O23
O41.1
O88.3
O98
O91

© 2023 Melhammar L et al. *JAMA Network Open.*

Organ dysfunction	
	I95.9
	R57.8
	R57.9
	R57.2
	J96.
	J80
	J98.4
	R06.0
	R06.8
	F05
	G93.1
	G93.4
	R40
	N17.
	N19
	E87.2
	D65
	D68.8
	D68.9
	D69.5
	D69.6
	K72.0
	K72.7
	K72.9
	K76.2
	K76.3

© 2023 Melhammar L et al. *JAMA Network Open.*

eTable 1. SOFA Score Adapted for Use Outside of Intensive Care

Organ system	SOFA score 1	2	3	4
**Respiration** PaO_2/FIO_2 mmHg<br>corresponding SpO_2 without O_2 support	< 400<br><95%	< 300<br><91%	<200<br>with respiratory support	<100<br>with respiratory support
**Coagulation** Platelets x 10^9/L	< 150	< 100	< 50	< 20
**Liver** Bilirubin µmol/L	>19	>32	>101	>204
**Cardiovascular** Hypotension	MAP < 70mmHg	Dopamine ≤ 5 µg/kg/min or dobutamine<br>for at least one hour	Dopamine > 5 or epinephrine or norepinephrine ≤ 0,1 µg/kg/min<br>for at least one hour	Dopamine > 15 or epinephrine or norepinephrine >0.1 µg/kg/min<br>for at least one hour
**Central Nervous System**<br>Glascow Coma Scale	13-14	10-12	6-9	3-5
**Renal** Creatinine µmol/L<br>or urine output mL/day	>110	>170	>300<br>< 500	>440<br>< 200

© 2023 Melhammar L et al. *JAMA Network Open.*

eTable 2. The Linder-Mellhammar Criteria of Infection

For each focus, 1-4 points are given for the following entities: symptoms and signs, radiological findings, laboratory findings, and microbiologic findings in relation to the suspected infection. These entities are grouped together as rows in a table for each infection focus. The highest scores from all rows are added together to give a total score for each focus. The level of evidence of infection is graded according to the scores:
0-1 points, no infection
2 points, possible infection
3 points, probable infection,
4 or more points, proven infection

© 2023 Melhammar L et al. *JAMA Network Open.*

Table 1. LOWER RESPIRATORY TRACT INFECTION

Clinical entity	1	2	3	4
**Symptoms**	Localized symptoms<br>• Purulent sputum or change in character of sputum<br>• New onset of cough or worsening of cough<br>• Pleural pain<br>Generalized symptoms<br>• Fever >38°C or history of fever<br>• Rigors<br>• Leukocytosis or leukopenia			
**Signs**	Possible fine crackles without other likely cause	Distinct crackles or bronchial breath sounds		
**Radiological findings**	Possible pneumonic infiltrate		Probable pneumonic infiltrate	Cavitation<br>Abscess<br>Empyema
**Microbiological findings**	• Detection of viral pathogens in respiratory tract<br>• Detection of pneumococci-antigen in urine	• Growth of likely pathogen from respiratory secretions<br>• Pneumocystis detected in immunocompromised patient<br>• Detection of legionella-antigen in urine	• Detection of Chlamydophila, Mycoplasma, Chlamydia, Legionella antigen, DNA or RNA in respiratory tract<br>• Growth of pathogens in specimen from bronchoscopy<br>• Growth in blood culture of pneumonia-pathogens without any other source in association with focal symptoms or signs	Detection of pathogens in pleural fluid culture, not from already present drain

© 2023 Melhammar L et al. *JAMA Network Open.*

Table 2. URINARY TRACT INFECTION

Clinical entity	1	2	3	4
**Symptoms**	Localized symptoms<br>• Mild urinary tract symptoms[1]<br>Generalized symptoms<br>• Fever >38°C or history of fever<br>• Rigors<br>• Leukocytosis or leukopenia	Distinct urinary tract symptoms[1]		
**Signs**	Localized tenderness (suprapubic or surrounding kidney)			
**Radiologic findings**				Abscess or inflammation
**Laboratory findings**	• Positive dipstick for leukocyte esterase or nitrate<br>• or pyuria in microscopic exam not from existing catheter			
**Microbiological findings**	$>10^5$ CFU/mL of a single primary urinary pathogen from existing catheter (>7 days)	$>10^5$ CFU/mL of a single pathogen not from existing catheter	Growth in blood culture of uropathogen without any other source	• Growth in other specimen than urine from the bladder or upper urinary tract<br>• Growth of same pathogen in blood culture and urine

1. Symptoms relevance graded by clinician, can include urgency, frequency, dysuria

© 2023 Melhammar L et al. *JAMA Network Open.*

Table 3. ABDOMINAL INFECTION

Clinical entity	1	2	3	4
**Symptoms and signs**	Localized symptoms and signs<br>• abdominal pain<br>• tenderness<br>• jaundice<br>Generalized symptoms and signs<br>• Fever >38°C or history of fever<br>• Rigors<br>• Leukocytosis or leucopoenia	Peritonitis		Surgical confirmation
**Radiological findings**		Inflammatory findings		Abscess Perforation
**Laboratory findings**		Inflammatory peritoneal fluid		
**Microbiological findings**			Growth in blood culture with pathogens compatible of abdominal infection in association with focal symptoms or signs	Detection in specimen from the affected site, not from remaining drain

Table 4. GASTROINTESTINAL INFECTION

Clinical entity	1	2	3	4
**Symptoms and signs**		Indistinct symptoms and signs[1]<br>Generalized symptoms and signs<br>• Fever >38°C or history of fever<br>• Rigors<br>• Leukocytosis or leukopenia<br>Localized symptoms	Distinct symptoms and signs[1]<br>Generalized symptoms and signs<br>• Fever >38°C or history of fever<br>• Rigors<br>• Leukocytosis or leukopenia<br>Localized symptoms	

© 2023 Melhammar L et al. *JAMA Network Open.*

		• Diarrhea<br>• Abdominal pain<br>• Tenderness<br>• Nausea<br>• Vomiting	• Diarrhea<br>• Abdominal pain<br>• Tenderness<br>• Nausea<br>• Vomiting	
**Microbiological findings**		Enteric pathogen or clostridial toxin detected from stool or rectal swab		Growth in blood culture without any other source with pathogens compatible of gastrointestinal infection[2]

1. Symptoms and signs relevance graded by clinician

© 2023 Melhammar L et al. *JAMA Network Open.*

Table 5. SKIN & SOFT-TISSUE INFECTION

Clinical entity	1	2	3	4
**Symptoms**	Localized symptoms<br>• Pain<br>• Tenderness<br>• Swelling<br>• Heat<br>Generalized symptoms<br>• Fever >38°C or history of fever<br>• Rigors<br>• Leukocytosis or leukopenia		Toxic shock (shock, generalized erythema w/wo vomiting) in association with focal symptoms or signs	
**Signs**			• Erythema (new localized)<br>• drainage of pus<br>• lymphangitis<br>• crepitations	Surgical confirmation
**Radiological findings**			Presence of inflammation or abscess	
**Laboratory findings**		Positive antigen test for group A streptococci from suspected site of infection		
**Microbiological findings**		Detection in superficial specimen from suspected focus of infection of relevant pathogens	Growth in blood culture without any other source with pathogens compatible of SSTI in association with focal symptoms or signs	Detection in specimen from the affected site, not from remaining drain or superficial specimen

© 2023 Melhammar L et al. *JAMA Network Open.*

Table 6. BONE AND JOINT INFECTION

Clinical entity	1	2	3	4
**Symptoms and signs**	Localized symptoms and signs<br>• Swelling<br>• Tenderness<br>• Limitation of motion<br>• Heat<br>Generalized symptoms<br>• Fever >38°C or history of fever<br>• Rigors<br>• Leukocytosis or leukopenia			Surgical confirmation
**Radiological findings**		Radiological evidence		
**Laboratory findings**		Joint fluid analysis compatible of infection, without other explanation		
**Microbiological findings**			Growth in blood culture without any other source with pathogens compatible of bone and joint infection in association with focal symptoms or signs	Detection in specimen from the affected site

© 2023 Melhammar L et al. *JAMA Network Open.*

Table 7. CENTRAL NERVOUS SYSTEM INFECTION

Clinical entity	1	2	3	4
**Symptoms and signs**	Localized symptoms and signs<br>• headache<br>• irritability<br>• mild neck stiffness<br>Generalized symptoms<br>• Fever >38°C or history of fever<br>• Rigors<br>• Leukocytosis or leukopenia	Distinct nuchal rigidity		Surgical confirmation
**Radiological findings**			Radiological evidence	Abscess
**Laboratory findings**			Increased white blood cells, protein and/or decreased glucose in CSF	
**Microbiological findings**			Growth in blood culture without any other source with pathogens compatible of CNS infection in association with focal symptoms or signs	Detection of pathogens in CSF<br>Growth in specimen from the affected site

© 2023 Melhammar L et al. *JAMA Network Open.*

Table 8. PRIMARY BLOODSTREAM INFECTIONS

Clinical entity	1	2	3	4
**Microbiological findings**				Growth in blood culture of pathogens without any other source of infection. Growth in blood culture with possible skin contaminants[1] the same in two or more cultures drawn from separate venous punctures

1. For example Coagulase-negative staphylococci, Propionibacterium cuti, Bacillus species, diphteroids or micrococci

Primary bloodstream infection is only considered when score is higher than in any other infection focus.

Table 9. CATHETER-RELATED INFECTION

Clinical entity	1	2	3	4
**Microbiological findings**		Growth in blood culture from catheter exit site		
**Microbiological findings**		Growth in culture of tip		
**Microbiological findings**		Growth in blood culture		

For a proven infection, the same microorganism needs to be found in different specimen

For a proven infection based on blood culture and blood culture from catheter, growth has to be recorded >120 minutes earlier from catheter

Table 10. EAR NOSE THROAT INFECTION

Clinical entity	1	2	3	4
**Symptoms**	Localized symptoms<br>• Pain<br>Generalized symptoms<br>• Fever >38°C or history of fever<br>• Rigors<br>• Leukocytosis or leukopenia			Surgical confirmation
**Signs**	Erythema of pharynx	• Purulent drainage<br>• Inflammation in the ear canal<br>• Retraction or decreased mobility of eardrum<br>• Visible abcess or swollen soft palate		
**Radiological findings**			Radiological evidence	
**Microbiological findings**	Positive antigen test for group A streptococci		• Detection in specimen from the affected site<br>• Growth in blood culture without any other source with pathogens compatible of ENT infection in association with focal symptoms or signs	

© 2023 Melhammar L et al. *JAMA Network Open.*

Table 11. UPPER RESPIRATORY TRACT INFECTION

Clinical entity	1	2	3	4
**Symptoms**	Localized symptoms<br>• Hoarseness<br>• New onset of cough or worsening of cough<br>• Purulent sputum<br>• Dyspnea<br>Generalized symptoms<br>• Fever >38°C or history of fever<br>• Rigors<br>• Leukocytosis or leukopenia			
**Signs**	Wheezing			
**Radiological findings**				
**Microbiological findings**		Detection of viral pathogens in respiratory tract		

Table 12. NEUTROPENIC FEVER

Clinical entity	1	2	3	4
**Symptoms, signs and findings**	Fever<br>>38°C sustained for 1 h<br>or repeatedly<br>or >38.3			
**Laboratory findings**	Neutrophil count <0.5 x 10^9/L			

© 2023 Melhammar L et al. *JAMA Network Open.*

Table 13. REPRODUCTIVE TRACT INFECTION

Clinical entity	1	2	3	4
**Symptoms and signs**	Localized symptoms<br>• Tenderness<br>• Dysuria<br>• Purulent discharge<br>Generalized symptoms<br>• Fever >38°C or history of fever<br>• Rigors<br>• Leukocytosis or leukopenia			Surgical confirmation
**Radiological findings**			Radiological evidence	Abscess
**Laboratory findings**		Positive antigen test for group A streptococci		
**Microbiological findings**			• Detection in specimen from the focus of infection<br>• Growth in blood culture without any other source with pathogens compatible of RTI infection, in association postpartum or with focal symptoms or signs	

© 2023 Melhammar L et al. *JAMA Network Open.*
