# Supplementary material for: Estimating Sepsis Incidence Using Administrative Data and Clinical Medical Record Review
Source: JAMA Netw Open. 2023 Aug 29;6(8):e2331168. doi: 10.1001/jamanetworkopen.2023.31168 (PMC10466163; doi:10.1001/jamanetworkopen.2023.31168)
Supplement: Supplement 2. — Data Sharing Statement [file jamanetwopen-e2331168-s002.pdf]

Data Sharing Statement

Mellhammar. Estimating Sepsis Incidence Using Administrative Data and Clinical Medical Record Review. *JAMA Netw Open*. Published August 29, 2023. doi:10.1001/jamanetworkopen.2023.31168

Data
Data available: Yes
Data types: Deidentified participant data
How to access data: Since the data can be regarded as sensitive (Health) data will be available upon request
When available: With publication

Supporting Documents
Document types: None

Additional Information
Who can access the data: Since the data can be regarded as sensitive (Health) data will be available upon request
Types of analyses: For further studies
Mechanisms of data availability: With investigator support
